# 2-[(*E*)-(4-Methyl­phen­yl)imino­meth­yl]-6-(morpholin-4-ylmeth­yl)phenol

**DOI:** 10.1107/S1600536810052311

**Published:** 2010-12-18

**Authors:** Mehmet Akkurt, Sevim Türktekin, Aliasghar Jarrahpour, Hashem Sharghi, Seid Ali Torabi Badrabady, Mahdi Aberi, Orhan Büyükgüngör

**Affiliations:** a Department of Physics, Faculty of Sciences, Erciyes University, 38039 Kayseri, Turkey; b Department of Chemistry, College of Sciences, Shiraz University, 71454 Shiraz, Iran; c Department of Physics, Faculty of Arts and Sciences, Ondokuz Mayıs University, 55139 Samsun, Turkey

## Abstract

In the title compound, C_19_H_22_N_2_O_2_, the morpholine ring adopts an almost perfect normal chair conformation with puckering parameters *Q*_T_, θ and ϕ of 0.5642 (18) Å, 177.32 (17) and ϕ = 10 (4)°, respectively. The two benzene rings make a dihedral angle of 42.67 (8)° with each other. An intra­molecular O—H⋯N hydrogen bond helps to stabilize the mol­ecular conformation. Aromatic C—H⋯π inter­actions and π–π stacking inter­actions [centroid–centroid distance = 3.6155 (15) Å] between the benzene rings contribute to the stabilization of the crystal structure.

## Related literature

For general background to Schiff bases with an azomethine or imine group (—C=N—), see: Akkurt *et al.* (2008 ▶); Dhar & Taploo (1982 ▶); Emregül *et al.* (2006 ▶); Jarrahpour & Khalili (2006 ▶); Jarrahpour *et al.* (2007 ▶); Mladenova *et al.* (2002 ▶); Przybylski *et al.* (2009 ▶); Sessler *et al.* (2006 ▶); Singh *et al.* (2006 ▶). For a similar structure, see: Akkurt *et al.* (2008 ▶). For reference structural data, see: Allen *et al.* (1987 ▶). For conformational analysis, see: Cremer & Pople (1975 ▶).
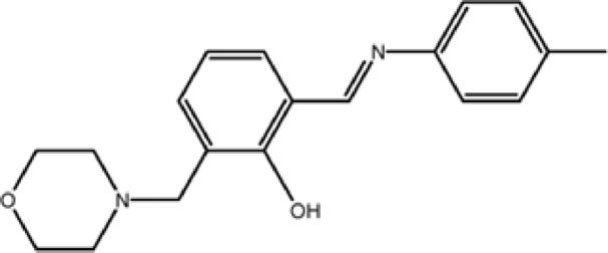


## Experimental

### 

#### Crystal data

C_19_H_22_N_2_O_2_
*M* *_r_* = 310.39
Triclinic, 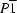

*a* = 9.807 (3) Å
*b* = 10.091 (3) Å
*c* = 10.528 (3) Å
α = 99.78 (2)°
β = 109.34 (2)°
γ = 115.18 (2)°
*V* = 828.7 (5) Å^3^
*Z* = 2
Mo *K*α radiation
μ = 0.08 mm^−1^
*T* = 296 K
0.53 × 0.40 × 0.23 mm

#### Data collection

Stoe IPDS 2 diffractometer
Absorption correction: integration (*X-RED32*; Stoe & Cie, 2002 ▶) *T*_min_ = 0.958, *T*_max_ = 0.982
15421 measured reflections
3443 independent reflections
2894 reflections with *I* > 2σ(*I*)
*R*_int_ = 0.026

#### Refinement

*R*[*F* ^2^ > 2σ(*F* ^2^)] = 0.042
*wR*(*F* ^2^) = 0.121
*S* = 1.08
3443 reflections
208 parameters
H-atom parameters constrained
Δρ_max_ = 0.19 e Å^−3^
Δρ_min_ = −0.17 e Å^−3^

### 

Data collection: *X-AREA* (Stoe & Cie, 2002 ▶); cell refinement: *X-AREA*; data reduction: *X-RED32* (Stoe & Cie, 2002 ▶); program(s) used to solve structure: *SIR97* (Altomare *et al.*, 1999 ▶); program(s) used to refine structure: *SHELXL97* (Sheldrick, 2008 ▶); molecular graphics: *ORTEP-3* (Farrugia, 1997 ▶); software used to prepare material for publication: *WinGX* (Farrugia, 1999 ▶).

## Supplementary Material

Crystal structure: contains datablocks global, I. DOI: 10.1107/S1600536810052311/si2317sup1.cif

Structure factors: contains datablocks I. DOI: 10.1107/S1600536810052311/si2317Isup2.hkl

Additional supplementary materials:  crystallographic information; 3D view; checkCIF report

## Figures and Tables

**Table 1:** Hydrogen-bond geometry (Å, °) *Cg*2 is the centroid of the C1–C6 ring.

*D*—H⋯*A*	*D*—H	H⋯*A*	*D*⋯*A*	*D*—H⋯*A*
O1—H1⋯N1	0.82	1.90	2.6261 (18)	147
C19—H19*A*⋯*Cg*2^i^	0.97	2.96	3.732 (3)	137

Symmetry code: (i) 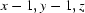.

**Fig. 1.**
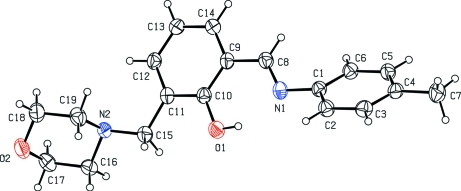
The title compound with the atom numbering scheme. Displacement ellipsoids for non-H atoms are drawn at the 30% probability level.

**Fig. 2.**
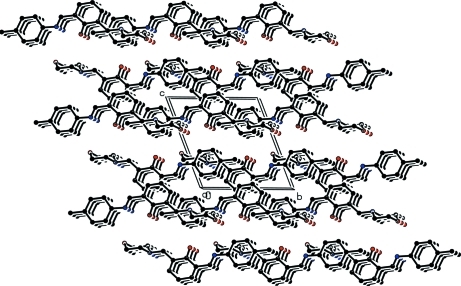
View of the packing of (I) down the a axis. All H atoms are omitted for clarity.

## supplementary crystallographic information

### Comment

Schiff bases, named for Hugo Schiff with the azomethine or imine group (—C═ N—) usually synthesized by condensation of a primary amine and an active carbonyl group under specific conditions. These compounds show a broad range of applications such as corrosion inhibitors (Emregül *et al.*, 2006), catalysts (Sessler *et al.*, 2006), pigments and polymer stabilizers. Schiff bases possess high biological activities including antibacterial (Jarrahpour *et al.*, 2006), antifungal (Singh *et al.*, 2006), antitumor (Mladenova *et al.*, 2002), antimalarial (Przybylski *et al.*, 2009), antiviral (Jarrahpour *et al.*, 2007) and antipyretic properties (Dhar *et al.*, 1982). Schiff bases are also good intermediates for the synthesis of other chemical substances such as 2-azetidinones.

As shown in Fig. 1, the morpholine ring (N2/O2/C16–C19) of the title compound (I) adopts a chair conformation with puckering parameters Q_T_, θ and φ of 0.5642 (18) Å, 177.32 (17) ° and φ = 10 (4) ° (Cremer & Pople (1975). The dihedral angle between the (C1–C6) and (C9–C14) benzene rings in (I) is 42.67 (8)°. The bond lengths exhibit normal values (Allen *et al.* 1987) and are comparable with those in our similar structure previously published (Akkurt *et al.*, 2008).

The molecular conformation of (I) is stabilized by intramolecular weak C12—H12···N2 and strong O1—H1···N1 hydrogen bonds (Table 1). In the crystal structure, a C—H···π interaction (Table 1) and a π-π stacking interaction between the C9–C14 benzene rings contribute to the stabilization of the crystal packing [*Cg*3···*Cg*3^ii^(symmetry code ii = 1 - *x*, 1 - *y*, -*z*) = 3.6155 (15) Å, where *Cg*3 is a centroid of the C9–C14 benzene ring]. Fig. 2 shows the crystal packing of (I) down the *a* axis.

### Experimental

Reaction of 2-hydroxy-3-(morpholinomethyl)benzaldehyde with 4-methylaniline in refluxing ethanol gave Schiff base (I) that recrystallized from ethanol to give orange crystals in 85% yield. [mp: 377–379 K]. IR (KBr, cm^-1^): 1615.2 (C═N), 3170.6–3310.5 (OH). ^1^H-NMR (250 MHz, CDCl_3_) δ (p.p.m): 2.30 (Me, s, 3H), 2.51 (CH_2_, t, 4H, *J* = 4.5), 3.60 (CH_2_, s, 2H), 3.69 (CH_2_, t, 4H, *J* = 4.5), 6.81–7.38 (m, ArH, 7H), 8.56 (HC═N, s, 1H), 13.68 (OH, s, 1H). ^13^C-NMR (CDCl_3_) δ (p.p.m): 21.0 (Me), 53.5 (N—CH_2_), 56.4 (CH_2_), 66.8 (O—CH_2_), 118.5–159.7 (C=C aromatic carbons), 161.3 (C=N).

### Refinement

All H atoms were placed at calculated positions and were treated as riding on their parent atoms with O—H = 0.82 Å, C—H = 0.93 (aromatic), 0.96(methyl) and 0.97 Å (methylene), and with *U*_iso_(H) = 1.5*U*_eq_(O,*C*) for hydroxy and methyl and *U*_iso_(H) = 1.2*U*_eq_(C) for aromatic, methylene.

### Crystal data

**Table:**

C_19_H_22_N_2_O_2_	*Z* = 2
*M**_r_* = 310.39	*F*(000) = 332
Triclinic, *P*1	*D*_x_ = 1.244 Mg m^−^^3^
Hall symbol: -P 1	Mo *K*α radiation, λ = 0.71073 Å
*a* = 9.807 (3) Å	Cell parameters from 2924 reflections
*b* = 10.091 (3) Å	θ = 2.2–28.0°
*c* = 10.528 (3) Å	µ = 0.08 mm^−^^1^
α = 99.78 (2)°	*T* = 296 K
β = 109.34 (2)°	Prism, yellow
γ = 115.18 (2)°	0.53 × 0.40 × 0.23 mm
*V* = 828.7 (5) Å^3^	

### Data collection

**Table:**

Stoe IPDS 2 diffractometer	3443 independent reflections
Radiation source: sealed X-ray tube, 12 x 0.4 mm long-fine focus	2894 reflections with *I* > 2σ(*I*)
plane graphite	*R*_int_ = 0.026
Detector resolution: 6.67 pixels mm^-1^	θ_max_ = 26.5°, θ_min_ = 2.2°
ω scans	*h* = −12→12
Absorption correction: integration (*X-RED32*; Stoe & Cie, 2002)	*k* = −12→12
*T*_min_ = 0.958, *T*_max_ = 0.982	*l* = −13→13
15421 measured reflections	

### Refinement

**Table:**

Refinement on *F*^2^	Primary atom site location: structure-invariant direct methods
Least-squares matrix: full	Secondary atom site location: difference Fourier map
*R*[*F*^2^ > 2σ(*F*^2^)] = 0.042	Hydrogen site location: inferred from neighbouring sites
*wR*(*F*^2^) = 0.121	H-atom parameters constrained
*S* = 1.08	*w* = 1/[σ^2^(*F*_o_^2^) + (0.0601*P*)^2^ + 0.1054*P*] where *P* = (*F*_o_^2^ + 2*F*_c_^2^)/3
3443 reflections	(Δ/σ)_max_ < 0.001
208 parameters	Δρ_max_ = 0.19 e Å^−^^3^
0 restraints	Δρ_min_ = −0.17 e Å^−^^3^

### Special details

**Table:**

Geometry. Bond distances, angles *etc*. have been calculated using the rounded fractional coordinates. All su's are estimated from the variances of the (full) variance-covariance matrix. The cell e.s.d.'s are taken into account in the estimation of distances, angles and torsion angles
Refinement. Refinement on *F*^2^ for ALL reflections except those flagged by the user for potential systematic errors. Weighted *R*-factors *wR* and all goodnesses of fit *S* are based on *F*^2^, conventional *R*-factors *R* are based on *F*, with *F* set to zero for negative *F*^2^. The observed criterion of *F*^2^ > σ(*F*^2^) is used only for calculating -*R*-factor-obs *etc*. and is not relevant to the choice of reflections for refinement. *R*-factors based on *F*^2^ are statistically about twice as large as those based on *F*, and *R*-factors based on ALL data will be even larger.

### Fractional atomic coordinates and isotropic or equivalent isotropic displacement parameters (Å^2^)

**Table:**

	*x*	*y*	*z*	*U*_iso_*/*U*_eq_	
O1	0.68415 (12)	0.65889 (11)	0.31759 (10)	0.0605 (3)	
O2	0.25927 (15)	−0.05310 (13)	0.37129 (14)	0.0731 (4)	
N1	0.70908 (14)	0.89705 (12)	0.23661 (12)	0.0509 (3)	
N2	0.39377 (13)	0.18806 (12)	0.26385 (12)	0.0470 (3)	
C1	0.80432 (16)	1.05345 (14)	0.24513 (14)	0.0460 (4)	
C2	0.90410 (18)	1.17249 (16)	0.38095 (15)	0.0529 (4)	
C3	1.00285 (17)	1.32583 (15)	0.39579 (15)	0.0541 (4)	
C4	1.00833 (16)	1.36570 (15)	0.27667 (15)	0.0508 (4)	
C5	0.90948 (17)	1.24557 (15)	0.14201 (15)	0.0515 (4)	
C6	0.80862 (16)	1.09138 (14)	0.12519 (14)	0.0492 (4)	
C7	1.1219 (2)	1.53245 (17)	0.29286 (19)	0.0719 (5)	
C8	0.56247 (17)	0.80217 (15)	0.13084 (15)	0.0496 (4)	
C9	0.46275 (16)	0.63836 (14)	0.11187 (14)	0.0464 (4)	
C10	0.52758 (16)	0.57282 (14)	0.20561 (13)	0.0461 (4)	
C11	0.43183 (16)	0.41315 (14)	0.18260 (14)	0.0469 (4)	
C12	0.27222 (17)	0.32268 (15)	0.06675 (15)	0.0503 (4)	
C13	0.20689 (17)	0.38570 (16)	−0.02683 (15)	0.0538 (4)	
C14	0.30183 (17)	0.54185 (16)	−0.00456 (15)	0.0529 (4)	
C15	0.51246 (18)	0.34623 (16)	0.28015 (16)	0.0565 (4)	
C16	0.48127 (18)	0.10773 (17)	0.31696 (16)	0.0555 (5)	
C17	0.3568 (2)	−0.05472 (17)	0.29914 (17)	0.0625 (5)	
C18	0.1757 (2)	0.0270 (2)	0.3227 (2)	0.0783 (7)	
C19	0.2971 (2)	0.19116 (18)	0.34212 (18)	0.0620 (5)	
H1	0.72840	0.74990	0.32090	0.0910*	
H2	0.90420	1.14840	0.46240	0.0630*	
H3	1.06710	1.40420	0.48720	0.0650*	
H5	0.91110	1.26940	0.06080	0.0620*	
H6	0.74370	1.01320	0.03360	0.0590*	
H7A	1.23740	1.56600	0.35230	0.0860*	
H7B	1.09230	1.60030	0.33720	0.0860*	
H7C	1.10810	1.53690	0.19930	0.0860*	
H8	0.51770	0.83940	0.06320	0.0600*	
H12	0.20700	0.21690	0.05110	0.0600*	
H13	0.09940	0.32250	−0.10420	0.0650*	
H14	0.25840	0.58370	−0.06780	0.0630*	
H15A	0.60110	0.34430	0.25960	0.0680*	
H15B	0.56460	0.41530	0.37980	0.0680*	
H16A	0.56130	0.16730	0.41820	0.0670*	
H16B	0.54360	0.10120	0.26380	0.0670*	
H17A	0.28230	−0.11600	0.19710	0.0750*	
H17B	0.41730	−0.10540	0.33660	0.0750*	
H18A	0.11260	0.03090	0.37590	0.0940*	
H18B	0.09590	−0.03110	0.22120	0.0940*	
H19A	0.23530	0.24150	0.30660	0.0740*	
H19B	0.37330	0.25170	0.44400	0.0740*	

### Atomic displacement parameters (Å^2^)

**Table:**

	*U*^11^	*U*^22^	*U*^33^	*U*^12^	*U*^13^	*U*^23^
O1	0.0572 (6)	0.0439 (5)	0.0533 (5)	0.0144 (4)	0.0117 (4)	0.0177 (4)
O2	0.0841 (8)	0.0696 (7)	0.0967 (8)	0.0459 (6)	0.0539 (7)	0.0551 (6)
N1	0.0570 (7)	0.0382 (5)	0.0558 (6)	0.0216 (5)	0.0276 (5)	0.0173 (5)
N2	0.0482 (6)	0.0411 (5)	0.0550 (6)	0.0236 (5)	0.0240 (5)	0.0224 (5)
C1	0.0463 (6)	0.0371 (6)	0.0536 (7)	0.0211 (5)	0.0225 (5)	0.0160 (5)
C2	0.0569 (8)	0.0476 (7)	0.0503 (7)	0.0247 (6)	0.0234 (6)	0.0174 (6)
C3	0.0514 (7)	0.0434 (7)	0.0511 (7)	0.0203 (6)	0.0157 (6)	0.0082 (5)
C4	0.0447 (7)	0.0383 (6)	0.0602 (8)	0.0189 (5)	0.0184 (6)	0.0156 (5)
C5	0.0531 (7)	0.0445 (7)	0.0542 (7)	0.0225 (6)	0.0237 (6)	0.0207 (6)
C6	0.0499 (7)	0.0396 (6)	0.0486 (7)	0.0193 (5)	0.0192 (6)	0.0120 (5)
C7	0.0678 (10)	0.0419 (7)	0.0777 (10)	0.0143 (7)	0.0231 (8)	0.0184 (7)
C8	0.0541 (7)	0.0432 (7)	0.0566 (7)	0.0257 (6)	0.0280 (6)	0.0218 (6)
C9	0.0490 (7)	0.0404 (6)	0.0527 (7)	0.0218 (5)	0.0269 (6)	0.0188 (5)
C10	0.0473 (7)	0.0406 (6)	0.0444 (6)	0.0186 (5)	0.0206 (5)	0.0138 (5)
C11	0.0502 (7)	0.0410 (6)	0.0506 (7)	0.0216 (5)	0.0256 (6)	0.0190 (5)
C12	0.0487 (7)	0.0384 (6)	0.0585 (8)	0.0177 (5)	0.0249 (6)	0.0183 (5)
C13	0.0436 (7)	0.0474 (7)	0.0583 (8)	0.0180 (6)	0.0174 (6)	0.0196 (6)
C14	0.0504 (7)	0.0511 (7)	0.0605 (8)	0.0268 (6)	0.0246 (6)	0.0271 (6)
C15	0.0509 (7)	0.0475 (7)	0.0601 (8)	0.0194 (6)	0.0192 (6)	0.0240 (6)
C16	0.0567 (8)	0.0581 (8)	0.0620 (8)	0.0354 (7)	0.0271 (7)	0.0282 (6)
C17	0.0816 (10)	0.0525 (8)	0.0645 (9)	0.0414 (8)	0.0325 (8)	0.0284 (7)
C18	0.0730 (10)	0.0819 (11)	0.1191 (15)	0.0480 (9)	0.0605 (11)	0.0659 (11)
C19	0.0761 (10)	0.0610 (8)	0.0774 (10)	0.0451 (8)	0.0470 (8)	0.0364 (7)

### Geometric parameters (Å, °)

**Table:**

O1—C10	1.3492 (18)	C16—C17	1.501 (2)
O2—C17	1.408 (3)	C18—C19	1.500 (3)
O2—C18	1.419 (3)	C2—H2	0.9300
O1—H1	0.8200	C3—H3	0.9300
N1—C8	1.277 (2)	C5—H5	0.9300
N1—C1	1.4189 (19)	C6—H6	0.9300
N2—C15	1.458 (2)	C7—H7A	0.9600
N2—C16	1.461 (2)	C7—H7B	0.9600
N2—C19	1.454 (3)	C7—H7C	0.9600
C1—C2	1.391 (2)	C8—H8	0.9300
C1—C6	1.3887 (19)	C12—H12	0.9300
C2—C3	1.378 (2)	C13—H13	0.9300
C3—C4	1.391 (2)	C14—H14	0.9300
C4—C7	1.511 (2)	C15—H15A	0.9700
C4—C5	1.388 (2)	C15—H15B	0.9700
C5—C6	1.383 (2)	C16—H16A	0.9700
C8—C9	1.453 (2)	C16—H16B	0.9700
C9—C10	1.407 (2)	C17—H17A	0.9700
C9—C14	1.395 (2)	C17—H17B	0.9700
C10—C11	1.402 (2)	C18—H18A	0.9700
C11—C15	1.513 (2)	C18—H18B	0.9700
C11—C12	1.383 (2)	C19—H19A	0.9700
C12—C13	1.388 (2)	C19—H19B	0.9700
C13—C14	1.373 (2)		
			
O1···N1	2.6261 (18)	H6···C8	2.7300
O2···N2	2.852 (2)	H6···H8	2.3300
O1···H15B	2.5400	H6···N2^ii^	2.9100
O1···H19B^i^	2.8300	H7C···H5	2.3500
O1···H15A	2.8200	H8···C6	2.6600
O2···H2^i^	2.7700	H8···H6	2.3300
N1···O1	2.6261 (18)	H8···H14	2.4300
N2···O2	2.852 (2)	H12···N2	2.5300
N1···H1	1.9000	H12···C19	2.9800
N2···H12	2.5300	H12···H19A	2.5700
N2···H6^ii^	2.9100	H13···H18B^vii^	2.5000
C1···C18^iii^	3.596 (3)	H14···H8	2.4300
C8···C12^ii^	3.409 (3)	H14···C5^vi^	2.8800
C10···C14^ii^	3.432 (3)	H15A···O1	2.8200
C12···C8^ii^	3.409 (3)	H15A···H16B	2.2800
C12···C19	3.368 (2)	H15B···O1	2.5400
C14···C10^ii^	3.432 (3)	H15B···H16A	2.5900
C18···C1^iv^	3.596 (3)	H15B···H19B	2.3000
C19···C12	3.368 (2)	H16A···H15B	2.5900
C1···H16B^v^	2.8600	H16A···H19B	2.4100
C1···H18A^iii^	3.0000	H16B···C1^viii^	2.8600
C2···H18A^iii^	2.9600	H16B···C2^viii^	3.0300
C2···H16B^v^	3.0300	H16B···H15A	2.2800
C3···H19A^iii^	3.1000	H17A···H18B	2.3900
C4···H19A^iii^	2.9400	H17A···H5^ii^	2.4600
C5···H14^vi^	2.8800	H18A···C1^iv^	3.0000
C6···H8	2.6600	H18A···C2^iv^	2.9600
C8···H1	2.4200	H18B···H17A	2.3900
C8···H6	2.7300	H18B···H13^vii^	2.5000
C11···H19A	2.8300	H19A···C3^iv^	3.1000
C12···H19A	2.8700	H19A···C4^iv^	2.9400
C19···H12	2.9800	H19A···C11	2.8300
H1···N1	1.9000	H19A···C12	2.8700
H1···C8	2.4200	H19A···H12	2.5700
H2···O2^i^	2.7700	H19B···H15B	2.3000
H5···H7C	2.3500	H19B···H16A	2.4100
H5···H17A^ii^	2.4600	H19B···O1^i^	2.8300
			
C17—O2—C18	110.04 (14)	C5—C6—H6	120.00
C10—O1—H1	109.00	C4—C7—H7A	109.00
C1—N1—C8	120.07 (13)	C4—C7—H7B	109.00
C15—N2—C19	111.36 (13)	C4—C7—H7C	109.00
C16—N2—C19	108.24 (13)	H7A—C7—H7B	110.00
C15—N2—C16	111.27 (14)	H7A—C7—H7C	109.00
N1—C1—C6	122.95 (12)	H7B—C7—H7C	109.00
C2—C1—C6	118.83 (13)	N1—C8—H8	119.00
N1—C1—C2	118.13 (12)	C9—C8—H8	119.00
C1—C2—C3	120.53 (13)	C11—C12—H12	119.00
C2—C3—C4	121.30 (13)	C13—C12—H12	119.00
C3—C4—C5	117.56 (14)	C12—C13—H13	120.00
C5—C4—C7	120.84 (14)	C14—C13—H13	120.00
C3—C4—C7	121.57 (13)	C9—C14—H14	120.00
C4—C5—C6	121.83 (13)	C13—C14—H14	120.00
C1—C6—C5	119.93 (13)	N2—C15—H15A	109.00
N1—C8—C9	122.64 (14)	N2—C15—H15B	109.00
C8—C9—C10	121.32 (13)	C11—C15—H15A	109.00
C8—C9—C14	119.69 (13)	C11—C15—H15B	109.00
C10—C9—C14	118.95 (13)	H15A—C15—H15B	108.00
O1—C10—C11	117.69 (13)	N2—C16—H16A	110.00
C9—C10—C11	120.53 (13)	N2—C16—H16B	110.00
O1—C10—C9	121.76 (13)	C17—C16—H16A	110.00
C10—C11—C12	118.40 (13)	C17—C16—H16B	110.00
C12—C11—C15	123.20 (13)	H16A—C16—H16B	108.00
C10—C11—C15	118.29 (13)	O2—C17—H17A	109.00
C11—C12—C13	121.68 (14)	O2—C17—H17B	109.00
C12—C13—C14	119.68 (15)	C16—C17—H17A	109.00
C9—C14—C13	120.76 (14)	C16—C17—H17B	109.00
N2—C15—C11	113.61 (14)	H17A—C17—H17B	108.00
N2—C16—C17	110.36 (15)	O2—C18—H18A	109.00
O2—C17—C16	112.22 (15)	O2—C18—H18B	109.00
O2—C18—C19	112.08 (17)	C19—C18—H18A	109.00
N2—C19—C18	110.12 (15)	C19—C18—H18B	109.00
C1—C2—H2	120.00	H18A—C18—H18B	108.00
C3—C2—H2	120.00	N2—C19—H19A	110.00
C2—C3—H3	119.00	N2—C19—H19B	110.00
C4—C3—H3	119.00	C18—C19—H19A	110.00
C4—C5—H5	119.00	C18—C19—H19B	110.00
C6—C5—H5	119.00	H19A—C19—H19B	108.00
C1—C6—H6	120.00		
			
C17—O2—C18—C19	−56.61 (19)	N1—C8—C9—C10	−3.8 (3)
C18—O2—C17—C16	56.19 (18)	N1—C8—C9—C14	178.64 (17)
C8—N1—C1—C6	−39.4 (3)	C10—C9—C14—C13	0.8 (3)
C8—N1—C1—C2	143.89 (18)	C14—C9—C10—C11	−0.2 (2)
C1—N1—C8—C9	176.53 (15)	C8—C9—C10—O1	0.5 (2)
C19—N2—C15—C11	−79.79 (16)	C14—C9—C10—O1	178.05 (15)
C15—N2—C16—C17	−180.00 (13)	C8—C9—C14—C13	178.35 (16)
C16—N2—C15—C11	159.35 (13)	C8—C9—C10—C11	−177.71 (15)
C15—N2—C19—C18	179.71 (14)	C9—C10—C11—C12	−0.6 (2)
C19—N2—C16—C17	57.33 (16)	O1—C10—C11—C15	−2.6 (2)
C16—N2—C19—C18	−57.68 (17)	O1—C10—C11—C12	−178.89 (15)
N1—C1—C6—C5	−177.41 (17)	C9—C10—C11—C15	175.65 (15)
N1—C1—C2—C3	178.14 (17)	C12—C11—C15—N2	−14.1 (2)
C2—C1—C6—C5	−0.7 (3)	C10—C11—C15—N2	169.80 (14)
C6—C1—C2—C3	1.3 (3)	C15—C11—C12—C13	−175.25 (16)
C1—C2—C3—C4	−1.2 (3)	C10—C11—C12—C13	0.8 (3)
C2—C3—C4—C5	0.6 (3)	C11—C12—C13—C14	−0.2 (3)
C2—C3—C4—C7	−177.38 (18)	C12—C13—C14—C9	−0.6 (3)
C3—C4—C5—C6	0.0 (3)	N2—C16—C17—O2	−57.79 (17)
C7—C4—C5—C6	177.97 (18)	O2—C18—C19—N2	58.52 (19)
C4—C5—C6—C1	0.1 (3)		

Symmetry codes: (i) −*x*+1, −*y*+1, −*z*+1; (ii) −*x*+1, −*y*+1, −*z*; (iii) *x*+1, *y*+1, *z*; (iv) *x*−1, *y*−1, *z*; (v) *x*, *y*+1, *z*; (vi) −*x*+1, −*y*+2, −*z*; (vii) −*x*, −*y*, −*z*; (viii) *x*, *y*−1, *z*.

### Hydrogen-bond geometry (Å, °)

**Table:**

Cg2 is the centroid of the C1–C6 ring.

**Table:**

*D*—H···*A*	*D*—H	H···*A*	*D*···*A*	*D*—H···*A*
O1—H1···N1	0.82	1.90	2.6261 (18)	147
C12—H12···N2	0.93	2.53	2.876 (2)	102
C19—H19A···Cg2^iv^	0.97	2.96	3.732 (3)	137

Symmetry codes: (iv) *x*−1, *y*−1, *z*.
